# Novel Red Phosphor of Gd^3+^, Sm^3+^ co-Activated Ag*_x_*Gd_((2−*x*)/3)−0.3−*y*_Sm*_y_*Eu^3+^_0.30_☐_(1−2*x*−2*y*)/3_WO_4_ Scheelites for LED Lighting

**DOI:** 10.3390/ma16124350

**Published:** 2023-06-13

**Authors:** Vladimir A. Morozov, Bogdan I. Lazoryak, Aleksandra A. Savina, Elena G. Khaikina, Ivan I. Leonidov, Alexey V. Ishchenko, Dina V. Deyneko

**Affiliations:** 1 Chemistry Department, Moscow State University, 119991 Moscow, Russia; morozov111vla@mail.ru (V.A.M.); bilazoryak@gmail.com (B.I.L.); 2 Skolkovo Institute of Science and Technology, 121205 Moscow, Russia; a.savina@skoltech.ru; 3 Baikal Institute of Nature Management, Siberian Branch, Russian Academy of Science, 670047 Ulan-Ude, Russia; 4 Institute of Solid State Chemistry, Ural Branch, Russian Academy of Sciences, 620990 Ekaterinburg, Russia; 5 NANOTECH Center, Ural Federal University, 620002 Ekaterinburg, Russia; 6 Laboratory of Arctic Mineralogy and Material Sciences, Kola Science Centre, Russian Academy of Sciences, 184209 Apatity, Russia

**Keywords:** incommensurately modulated structures, white light-emitting diodes, scheelite, luminescence, phosphors, tungstates, silver, gadolinium, samarium, europium

## Abstract

Gd^3+^ and Sm^3+^ co-activation, the effect of cation substitutions and the creation of cation vacancies in the scheelite-type framework are investigated as factors influencing luminescence properties. Ag*_x_*Gd_((2−*x*)/3)−0.3−*y*_Sm*_y_*Eu^3+^_0.3_☐_(1−2*x*)/3_WO_4_ (*x* = 0.50, 0.286, 0.20; *y* = 0.01, 0.02, 0.03, 0.3) scheelite-type phases (A*_x_*GS*_y_*E) have been synthesized by a solid-state method. A powder X-ray diffraction study of A*_x_*GS*_y_*E (*x* = 0.286, 0.2; *y* = 0.01, 0.02, 0.03) shows that the crystal structures have an incommensurately modulated character similar to other cation-deficient scheelite-related phases. Luminescence properties have been evaluated under near-ultraviolet (n–UV) light. The photoluminescence excitation spectra of A*_x_*GS*_y_*E demonstrate the strongest absorption at 395 nm, which matches well with commercially available UV-emitting GaN-based LED chips. Gd^3+^ and Sm^3+^ co-activation leads to a notable decreasing intensity of the charge transfer band in comparison with Gd^3+^ single-doped phases. The main absorption is the ^7^F_0_ → ^5^L_6_ transition of Eu^3+^ at 395 nm and the ^6^H_5/2_ → ^4^F_7/2_ transition of Sm^3+^ at 405 nm. The photoluminescence emission spectra of all the samples indicate intense red emission due to the ^5^D_0_ → ^7^F_2_ transition of Eu^3+^. The intensity of the ^5^D_0_ → ^7^F_2_ emission increases from ~2 times (*x* = 0.2, *y* = 0.01 and *x* = 0.286, *y* = 0.02) to ~4 times (*x* = 0.5, *y* = 0.01) in the Gd^3+^ and Sm^3+^ co-doped samples. The integral emission intensity of Ag_0.20_Gd_0.29_Sm_0.01_Eu_0.30_WO_4_ in the red visible spectral range (the ^5^D_0_ → ^7^F_2_ transition) is higher by ~20% than that of the commercially used red phosphor of Gd_2_O_2_S:Eu^3+^. A thermal quenching study of the luminescence of the Eu^3+^ emission reveals the influence of the structure of compounds and the Sm^3+^ concentration on the temperature dependence and behavior of the synthesized crystals. Ag_0.286_Gd_0.252_Sm_0.02_Eu_0.30_WO_4_ and Ag_0.20_Gd_0.29_Sm_0.01_Eu_0.30_WO_4_, with the incommensurately modulated (3 + 1)D monoclinic structure, are very attractive as near-UV converting phosphors applied as red-emitting phosphors for LEDs.

## 1. Introduction

Light-emitting diodes (LEDs) have now mostly replaced the other light sources such as incandescent or fluorescent lamps. These new light sources are more reliable, last longer and are more environmentally friendly [1]. New light sources find interesting areas of application, for example, for architectural lighting, lighting in electronic technology, etc. [2].

Phosphors based on tungstates and molybdates with broad and intense absorption bands in the near UV region are widely used. Scheelite-type tungstates and molybdates doped with rare-earth elements are promising materials for WLED [3,4,5,6,7,8,9,10,11] and solid-state lasers [12,13,14,15]. For example, for a phosphor based on NaEu(WO_4_)_2_, the emission intensity at ~615 nm and the integral intensity are 8.5 and 5.0 times higher, respectively, than for commercial Y_2_O_2_S:Eu^3+^ phosphor (~626 nm) [4]. Such materials can determine temperature gradients with high accuracy, which is important for their application as thermographic phosphors [16,17,18,19].

In the scheelite-related phases Ag*_x_*Eu_(2−*x*)/3_☐_(1−2*x*)/3_WO_4_ and Ag*_x_*Gd_(2−*x*)/3−0.3_Eu_0.3_☐_(1−2*x*)/3_WO_4_, strong absorption occurs at 395 nm [20]. This absorption region is in good agreement with the absorption region of a commercial GaN-based LED chip emitting n–UV. The good agreement between the absorption regions of the studied tungstates and the GaN-based chip makes it possible to use these phosphors as emitters. Phosphor Ag*_x_*Gd_((2−*x*)/3)−0.3_Eu^3+^_0.3_☐_(1−2*x*)/3_WO_4_ is a more efficient emitter than Ag*_x_*Eu_(2−*x*)/3_☐_(1−2*x*)/3_WO_4_ [20].

In the present research, we have focused on Ag*_x_*Gd_((2−*x*)/3)−0.3−*y*_Sm*_y_*Eu^3+^_0.30_☐_(1−2*x*−2*y*)/3_WO_4_ scheelites to analyze the influence of Gd^3+^ and Sm^3+^ co-activation and the number of cation vacancies on the luminescent properties and structure. Sm^3+^ cation is usually used as a sensitizer to enhance the luminescence of Eu^3+^ cations [4,21,22,23]. By changing the concentration of Sm^3+^ cations, it is possible to increase the width of the excitation band and thereby increase the luminescence intensity of Eu^3+^ cations upon excitation of the blue LED or near UV.

## 2. Materials and Methods

### 2.1. Sample Preparation

Ag*_x_*Gd_((2−*x*)/3)−0.3−*y*_Sm*_y_*Eu^3+^_0.30_☐_(1−2*x*−2*y*)/3_WO_4_ (*x* = 0.5, 0.286, 0.2; *y* = 0.01–0.3) (A*_x_*GS*_y_*E—short entry) was synthesized from a stoichiometric mixture of *R*_2/3_☐_1/3_WO_4_ (*R* = Sm, Eu, Gd) at 833 K for 10 h, followed by annealing at 1283 K for 95 h by the solid phase method. Ag_2_WO_4_ was synthesized at 633 K for 12 h from stoichiometric mixtures of WO_3_ (99.99%) and AgNO_3_ (99.99%), followed by annealing at 833 K for 42 h. *R*_2/3_☐_1/3_WO_4_ tungstates were synthesized from stoichiometric mixtures WO_3_ and *R*_2_O_3_ (99.99%) at 883 K for 12 h, followed by annealing at 1133 K for 82 h. To study the luminescent properties of Ag*_x_R*_((2−*x*)/3)−0.3_Eu^3+^_0.30_☐_(1−2*x*)/3_WO_4_ (*R* = Eu, Gd; *x* = 0.5, 0.286, 0.2), we used samples obtained earlier [19,20].

### 2.2. Characterization

X-ray diffraction (XRD) patterns of A*_x_*GS*_y_*E (*x* = 0.5, 0.286, 0.2; *y* = 0.01–0.3) were obtained on a Thermo ARL X’TRA powder diffractometer (Waltham, MA, USA) (CuK_α_ radiation, λ = 1.5418 Å, Bragg–Brentano geometry, scintillation detector, at T_R_, the 5–70° 2*θ* range, steps 0.02°). Moreover, XRD patterns of Ag*_x_R*_((2−*x*)/3)−0.3_Eu^3+^_0.30_☐_(1−2*x*)/3_WO_4_ (*R* = Eu, Gd; *x* = 0.5, 0.286, 0.2) were obtained on a Huber G670 Guinier diffractometer (Rimsting, Germany) (Cu K_α1_ radiation, transmission mode, curved Ge(111) monochromator, image plate detector, at T_R_, HUBER Diffraktionstechnik GmbH & Co. (Rimsting, Germany), 4–100° 2θ range, steps 0.01°). The lattice parameters were refined by the Le Bail method [24] using JANA2006 software (version Jana2006 for Windows) [25].

Photoluminescence excitation (PLE) and emission (PL) spectra were obtained on a Varian Cary Eclipse fluorescence spectrometer (75 kW xenon light source, pulse τ = 2 μs, frequency *ν* = 80 Hz, resolution 0.5 nm; R928 PMT Hamamatsu, Hamamatsu Photonics, Hamamatsu, Japan). All spectrum measurements were performed at room temperature in a copper cell (∅ 10 mm × 20 mm) and corrected for the sensitivity of the spectrometer. The integral intensity (*I*_int_) of the ^5^D_0_ → ^7^F_2_ transition in polycrystalline Ag_0.50_Eu_0.50_WO_4_ was taken as 100%. The integral intensities of the ^5^D_0_ → ^7^F_2_ transition for other samples were normalized to the integral intensity of this transition for Ag_0.50_Eu_0.50_WO_4_. The Perkin Elmer LS–55 fluorescence spectrometer (PerkinElmer, Inc., Waltham, MA, USA) [26] was used to study the effect of temperature (300 K–700 K) on luminescent properties. The PL spectra were obtained with excitation at 395 nm, measurement step 20 s, heating rate 4 K/min. Before measuring the temperature, the presence of thermostimulated luminescence was checked upon excitation at 395 nm. All measurements of the samples were carried out under the same conditions.

## 3. Results

### 3.1. Crystallography 

**Figure 1:**
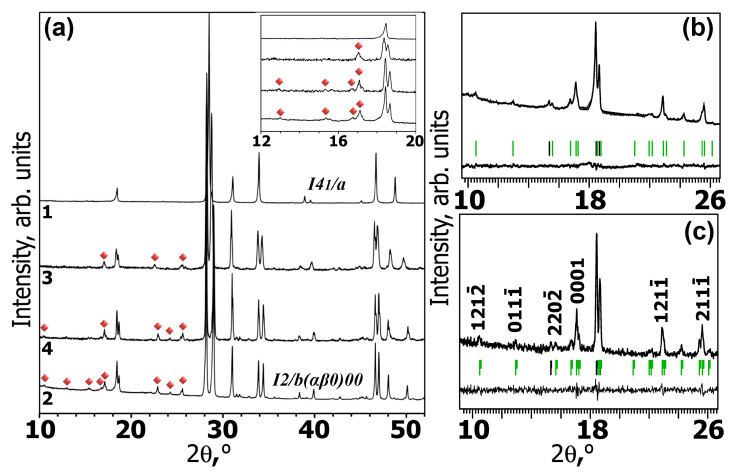
(**a**) Parts of PXRD patterns of the Ag_0.5_Gd_0.2_Eu_0.3_WO_4_ (1), Ag_0.2_Gd_0.3_Eu^3+^_0.3_☐_0.2_WO_4_ (2) and Ag*_x_*Gd_((2−*x*)/3)−0.33_Sm_0.03_Eu^3+^_0.30_☐_(1−2*x*−0.06)/3_WO_4_ (*x* = 0.286 (3), 0.2 (4)) samples in 2θ ranges of 10–52°. The satellite reflections are indicated with red diamonds; (**b**,**c**) parts of experimental, calculated and difference XPD profiles in 2θ ranges of 9.6–26.3° after Le Bail decomposition of Ag_0.2_Gd_0.3_Eu^3+^_0.3_☐_0.2_WO_4_ ((**b**) Huber G670) and Ag_0.2_Gd_0.27_Sm_0.03_Eu^3+^_0.30_☐_0.2_WO_4_ ((**c**) Thermo ARL X’TRA, Thermo Fisher Scientific, Waltham, MA, USA). Black and green bars mark the positions of the main and satellite reflections, respectively. The indexation of some satellite reflections is shown.

Figure 1 shows the XRD patterns of the four investigated phases. The *x* = 0.5 compounds, Ag_0.5_Gd_0.2−*y*_Sm*_y_*Eu^3+^_0.30_WO_4_ and Ag_0.5_Eu_0.2_Eu_0.3_WO_4_ belong to the tetragonal structure (space group (SG) *I*4_1_/*a*). The XRD patterns of A*_x_*GS*_y_*E (*x* = 0.286, 0.2; *y* = 0.01–0.3) and Ag_0.2_Eu_0.3_Eu^3+^_0.3_☐_0.2_WO_4_ show intense reflections of a scheelite-type superstructure and satellite reflections with low intensity. The patterns can be attributed to monoclinic incommensurately modulated (3 + 1)D symmetry (superspace group (SSG) *I*2/*b*(αβ0)00). The unit cell parameters of the A*_x_*GS*_y_*E samples, determined from X-ray diffraction patterns using the Le Bail decomposition in the SSG *I*2/*b*(αβ0)00 and the modulation vector **q**, are given in Table 1 together with reference data.

### 3.2. Luminescence Properties

**Figure 2:**
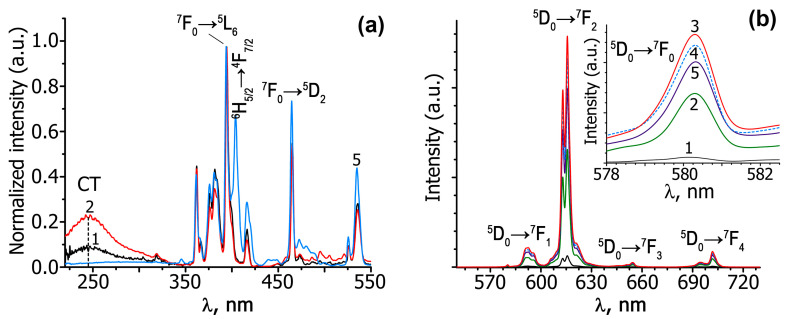
Excitation (λ_em_ = 615 nm) (**a**) and luminescence (λ_ex_ = 395 nm) (**b**) spectra of Ag_0.20_Eu_0.60_☐_0.20_WO_4_ (1) and Ag_0.20_Gd_0.30−*y*_Sm*_y_*Eu_0.30_☐_0.20_WO_4_ (*y* = 0 (2), 0.01 (3), 0.02 (4), 0.03 (5)) at room temperature. The luminescence spectra of the ^5^D_0_ → ^7^F_0_ transition for Eu^3+^ are shown in the inset (**b**).

**Figure 3:**
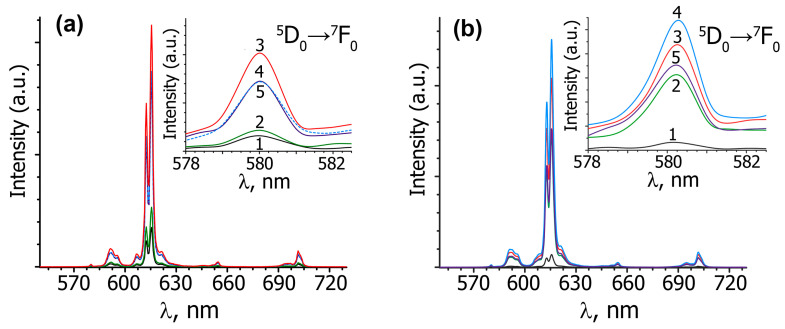
The luminescence spectra (λ_ex_ = 395 nm) of Ag_0.5_*R*_0.20−*y*_Sm*_y_*Eu_0.30_WO_4_ (**a**) and Ag_0.286_*R*_0.271−*y*_Sm*_y_*Eu_0.30_☐_0.143_WO_4_ (**b**) (*R* = Eu and *y* = 0 (1), *R* = Gd and *y* = 0 (2); *R* = Gd and *y* = 0.01 (3), 0.02 (4), 0.03 (5)) at room temperature. The luminescence spectra of the ^5^D_0_ → ^7^F_0_ transition for Eu^3+^ are shown in the inset (**a**,**b**).

The photoluminescence emission (PL) and excitation (PLE) spectra of A*_x_*GS*_y_*E (*x* = 0.5, 0.286, 0.2; *y* = 0–0.03) tungstates are shown in Figure 2 and Figure 3. Figure 2a shows PLE spectra for the Eu^3+^ in Ag_0.20_Gd_0.30−*y*_Sm*_y_*Eu_0.30_☐_0.20_WO_4_ (*y* = 0–0.03) upon emission at 615 nm. The excitation spectra (PLE) contain a broad band (220–320 nm) and a group of narrow lines (320–550 nm). The luminescence spectra of A*_x_*GS*_y_*E (*x* = 0.5, 0.286, 0.2; *y* = 0–0.03) (λ_ex_ = 395 nm) are shown in Figure 2b and Figure 3.

**Figure 4:**
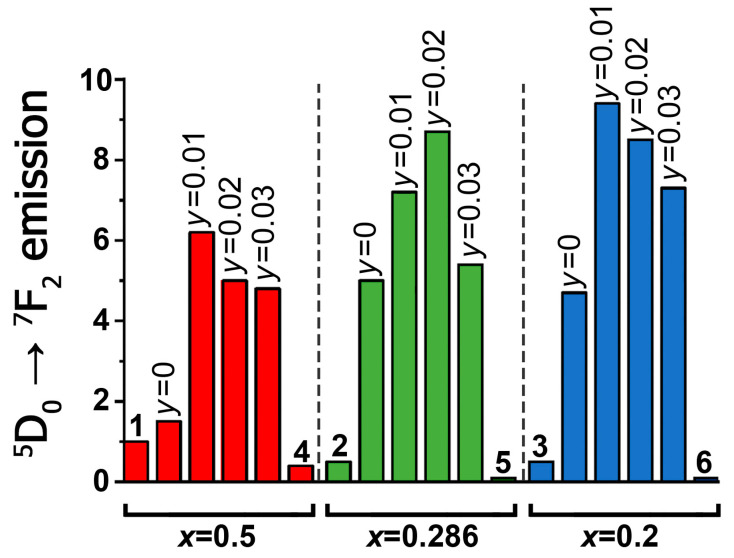
Change in the integrated luminescence intensity of the ^5^D_0_ → ^7^F_2_ transition in Eu^3+^ (λ_ex_ = 395 nm) for scheelite-related Ag*_x_*Eu^3+^_(2−*x*)/3_☐_(1−2*x*)/3_WO_4_ (*x* = 0.50 (1), 0.286 (2), 0.20 (3)), Ag*_x_*Sm_(2−*x*)/3−0.3_Eu_0.3_☐_(1−2*x*)/3_WO_4_ (*x* = 0.50 (4), 0.286 (5), 0.20 (6)) and Ag*_x_*Gd_((2−*x*)/3)−0.3−*y*_Sm*_y_*Eu^3+^_0.3_☐_(1−2*x*)/3_WO_4_ (*x* = 0.50, 0.286, 0.20; *y* = 0, 0.01, 0.02, 0.03). All intensities of the investigated phases are normalized to the value of the integral intensity of Ag_0.50_Eu_0.50_WO_4_ (1).

**Figure 5:**
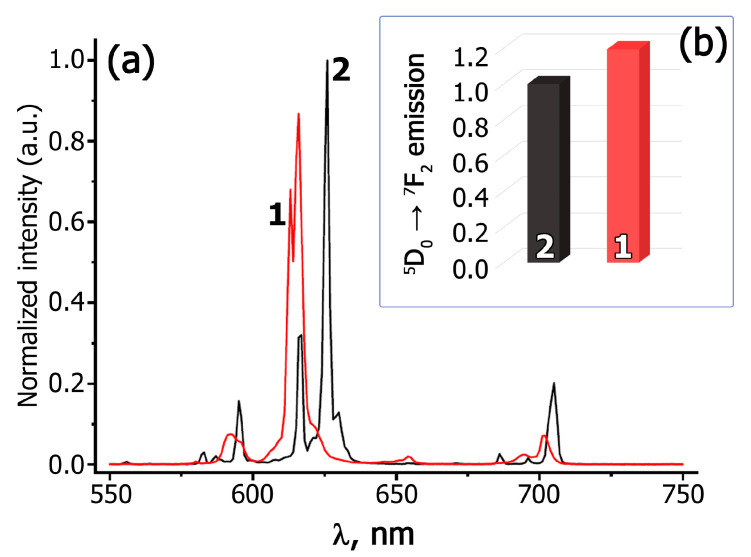
Parts of room temperature PL (λ_ex_ = 395 nm) spectra (**a**) and integral ^5^D_0_ → ^7^F_2_ emission intensity (**b**) of Ag_0.20_Gd_0.29_Sm_0.01_Eu_0.30_WO_4_ (1) and the commercially used phosphor Gd_2_O_2_S:Eu^3+^ (2). The luminescence (PL) spectra are normalized to the integral luminescence intensity of Gd_2_O_2_S:Eu^3+^.

The comparative integral intensity of the ^5^D_0_ → ^7^F_2_ luminescence for Ag*_x_*Eu^3+^_(2−*x*)/3_☐_(1−2*x*)/3_WO_4_ and A*_x_*GS*_y_*E scheelite-type phases is presented in Figure 4. The electric dipole transition ^5^D_0_ →^7^F_2_ at ~615 nm is the most intensive for all the luminescence spectra. The data on the effect of Gd^3+^ and Sm^3+^ co-activation on the ^5^D_0_ →^7^F_2_ transition luminescence intensity in Ag*_x_R*_((2−*x*)/3)−0.3−*y*_Sm*_y_*Eu^3+^_0.3_☐_(1−2*x*)/3_WO_4_ (*R* = Eu, Gd, Sm; *x* = 0.50, 0.286, 0.20; *y* = 0–0.03) are summarized in Table S1 (Supporting Information). The comparison of the PL spectra and integral ^5^D_0_ → ^7^F_2_ emission intensity of Ag_0.20_Gd_0.29_Sm_0.01_Eu_0.30_WO_4_ and the commercially used Gd_2_O_2_S:Eu^3+^ red phosphor is shown in Figure 5.

**Figure 6:**
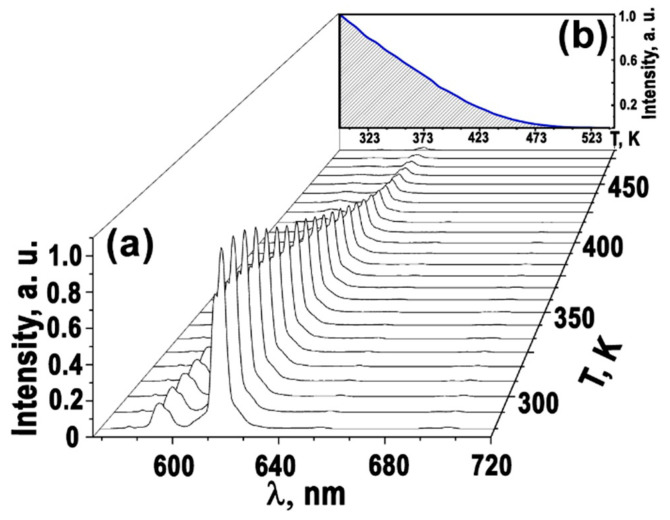
Variation of PL spectra (**a**) and luminescence intensity of the ^5^D_0_ → ^7^F_2_ transition as a function of temperature—blue line (**b**) for Ag_0.20_Gd_0.29_Sm_0.01_Eu_0.30_☐_0.20_WO_4_ at ~615 nm.

**Figure 7:**
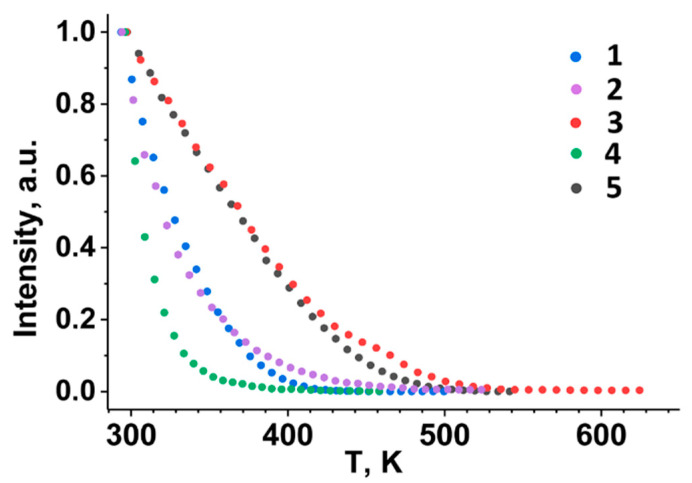
The temperature dependences of the ^5^D_0_ → ^7^F_2_ luminescence intensity at ~615 nm of Ag_0.5_*R*_0.20_Eu_0.30_WO_4_ (*R* = Gd (1), Sm (2)), Ag_0.20_*R*_0.30_Eu_0.30_☐_0.20_WO_4_ (*R* = Gd (3), Sm (4)) and Ag_0.20_Gd_0.29_Sm_0.01_Eu_0.30_☐_0.20_WO_4_ (5).

To evaluate the behavior of thermal quenching, the PL spectra were studied at temperatures ranging from 300 to 700 K. Figure 6 and Figure 7 show the temperature-dependent PL spectra and the intensity of the ^5^D_0_ → ^7^F_2_ transition at ~615 nm.

## 4. Discussion

The present work is focused on the scheelite-type phases of A*_x_*GS*_y_*E (*x* = 0.5, 0.286, 0.2; *y* = 0.01, 0.02, 0.03, 0.3) with a variable composition. One can establish that the substitution of Ag^+^ by *R*^3+^ (*R* = Eu, Gd, Sm) leads to a noticeable distortion of the scheelite-like framework. The phase with *x* = 0.5 crystallizes in a tetragonal scheelite-like structure (*I*4_1_/*a*). From the high-resolution synchrotron XRD data [20] for a sample with *x* = 0.5, it is found that there is no Ag^+^ and *R*^3+^ cations ordering.

We have previously analyzed that the formation of cationic vacancies in Ag*_x_*Gd_((2−*x*)/3)−0.3_Eu^3+^_0.3_☐_(1−2*x*)/3_WO_4_ [20] and Ag*_x_*Sm_(2−*x*)/3_☐_(1−2*x*)/3_WO_4_ [19] structures is accompanied by ordering of cations and vacancies with the formation of (3 + 1) D incommensurably modulated structures for Ag*_x_R*_(2−*x*)/3_☐_(1−2*x*)/3_WO_4_ (*R* = Eu, Sm; *x* = 0.286, 0.2) phases. As revealed earlier for Ag*_x_*Gd_((2−*x*)/3)−0.3_Eu^3+^_0.3_☐_(1−2*x*)/3_WO_4_ (*x* = 0.286, 0.2) [20] and shown here for A*_x_*GS*_y_*E (*x* = 0.286, 0.2; *y* = 0.01, 0.02, 0.03), the structures of these scheelite-related phases have an incommensurately modulated character too. The Ag*_x_R*_(2−*x*)/3_☐_(1−2*x*)/3_WO_4_ (*R* = Eu, Sm; *x* = 0.286, 0.2) aperiodic structures are built from two types of columns [… – (*R*O_8_/AgO_8_)–WO_4_ – …] and [… – ☐ – WO_4_ – …], which are extended along the *c*-axis. The structures of these phases are characterized by different distributions of Ag^+^/*R*^3+^ cations and vacancies in the A-cationic column of the scheelite structure. The specific coefficients *α* and *β* of the modulation vector **q** = *α*a* + *β*b* in incommensurately modulated structures, and the individual parameters of the atomic domains, define specific cations ordering for each modulated structure. In Ag*_x_R*_(2−*x*)/3_☐_(1−2*x*)/3_WO_4_ (*R* = Eu, Sm; *x* = 0.286, 0.2) structures, two types of *R*–aggregates have been described: [*R*_2_O_14_] dimers and infinite [*R*O_8_]*_n_* chains from EuO_8_ polyhedra [19,20].

**Figure 8:**
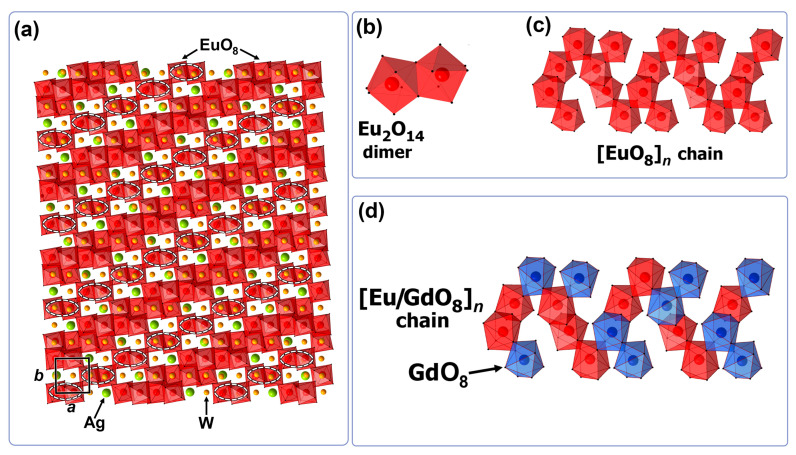
*ab*-projection of a part of the supercell (8*a* × 10*b* × 1*c*) of the aperiodic structure Ag_0.157_Eu_0.614_☐_0.229_WO_4_ refined in Ref. [20] (**a**). The Ag, W and O atoms are shown in green, yellow and red, respectively. AgO_8_ polyhedra and WO_4_ tetrahedra are not shown. [Eu_2_O_14_] dimers are marked with white ellipses. Shown are [Eu_2_O_14_] dimers (**b**) and [EuO_8_]*_n_* chains (**c**) of EuO_8_ polyhedra in the Ag_0.157_Eu_0.614_☐_0.229_WO_4_ and [Eu/GdO_8_]*_n_* chains (**d**) in the Ag*_x_*Gd_((2−*x*)/3)−0.3_Eu^3+^_0.3_☐_(1−2*x*)/3_WO_4_ structure.

As an example, a portion of the Ag_0.157_Eu_0.614_☐_0.229_WO_4_ disordered structure is shown in the [001] projection in Figure 8a. [Eu_2_O_14_]-dimers are Eu-pairs surrounded by only WO_4_ tetrahedra and isolated from all other Eu^3+^ in the infinite [EuO_8_]*_n_* chains by vacancies and Ag^+^ cations (Figure 8b,c). The amount of [Eu_2_O_14_]-dimers in the Ag*_x_*Eu_(2−*x*)/3_☐_(1−2*x*)/3_WO_4_ framework depends on *x*. In the structures of Ag*_x_*Eu_(2−*x*)/3_☐_(1−2*x*)/3_WO_4_, the number of Eu^3+^ cations involved in the formation of [Eu_2_O_14_]-dimers increases from 15.56% to 28.33%, with decreasing *x* from 0.238 to 0.157 [20]. There is a relationship between the number of [Eu_2_O_14_]-dimers in the structure and the luminescence characteristics (total ($Q_{L}^{Eu}$) and intrinsic ($Q_{Eu}^{Eu}$) quantum yields and lifetimes of Eu^3+^ (^5^D_0_) (τ_obs_)) [27]. The values of the luminescent characteristics increase with an increase in the number of Eu^3+^ dimers. However, the maximal of the luminescence characteristics is observed in the disordered structure of Na_0.5_Eu_0.5_MoO_4_.

The phosphors Ag*_x_*Gd_((2−*x*)/3)−0.3_Eu^3+^_0.3_☐_(1−2*x*)/3_WO_4_ at the values of *x* (0.5–0) have better characteristics for emitters than Ag*_x_*Eu_(2−*x*)/3_☐_(1−2*x*)/3_WO_4_ [20]. In Ag*_x_*Gd_((2−*x*)/3)−0.3_Eu^3+^_0.3_☐_(1−2*x*)/3_WO_4_ the emission of Eu^3+^ (λ_ex_ = 395 nm) increases with the Gd^3+^ content from 0.2 (*x* = 0.5) to 0.3 (*x* = 0.2) by a factor of 3 compared to the phosphors Ag*_x_*Eu_(2−*x*)/3_☐_(1−2*x*)/3_WO_4_ (Figure 4). The replacement of Eu^3+^ cations by Gd^3+^ is accompanied by the destruction of infinite [EuO_8_]*_n_* chains and the formation of [Eu_2_O_14_]-dimers and/or isolated EuO_8_ polyhedra inside them (Figure 8d).

The PLE spectra of Ag*_x_*Gd_((2−*x*)/3)−0.3_Eu^3+^_0.3_☐_(1−2*x*)/3_WO_4_ and Ag*_x_*Eu_(2−*x*)/3_☐_(1−2*x*)/3_WO_4_ are characterized by broad (220–320 nm) and narrow (320–500 nm) excitation bands. The broad excitation line peaking at ~240 nm is due to charge transfer (CT) from the 2*p* orbital of oxygen to the 3*d* orbital of tungsten in the WO_4_^2−^ tetrahedron [28,29] and coincides with the O^2−^ → Eu^3+^ CT band. Co-activation with Gd^3+^ and Sm^3+^ ions leads to a noticeable decrease in intensity of CT bands in comparison with Gd^3+^ single-doped phosphors (Figure 2a). All the phases reveal characteristic lines in the spectral region from 320 to 500 nm, which originate from 4*f*–4*f* transitions of Eu^3+^ and Sm^3+^ (for Sm-containing compounds). The main absorption bands in the PLE spectra are associated with the ^7^F_0_ → ^5^L_6_ Eu^3+^ transition at 395 nm and the ^6^H_5/2_ → ^4^F_7/2_ Sm^3+^ transition at 405 nm.

In the PL spectra of Ag*_x_R*_((2−*x*)/3)−0.3_Eu^3+^_0.30_☐_(1−2*x*)/3_WO_4_ (*R* = Eu, Gd; *x* = 0.5, 0.286, 0.2) and A*_x_*GS*_y_*E (*x* = 0.5, 0.286, 0.2; *y* = 0.01, 0.02, 0.03) in the region of 570–650 nm, the characteristic lines are observed for Eu^3+^ cations due to the ^5^D_0_ → ^7^F*_J_* (*J* = 0, 1, 2) transitions (Figure 2b and Figure 3). In the luminescence spectra of all phases, the most intense electric dipole transition ^5^D_0_ → ^7^F_2_ is observed at ~615 nm, which indicates the absence of a center of symmetry in the polyhedra of Eu^3+^ cations [30,31]. The magnetic dipole transition ^5^D_0_ → ^7^F_1_ is observed at 590 nm. The intensity of this transition depends a little on the environment of the luminescent cation, since it has a magnetic dipole nature. The asymmetry coefficient (R/O), *I_int_*(^5^D_0_ → ^7^F_2_)/*I_int_*(^5^D_0_ → ^7^F_1_) [32] is usually used to estimate the symmetry of the Eu^3+^ polyhedron. The high values of the R/O ratio for the studied tungstates (Figure 2 and Figure 3) indicate a non-centrosymmetric local environment of Eu^3+^ in these phases. Further evidence of the non-centrosymmetric environment of europium cations in the PL structure of A*_x_*GS*_y_*E (*x* = 0.5, 0.286, 0.2; *y* = 0.01, 0.02, 0.03) is the presence of the ^5^D_0_ → ^7^F_0_ transition band at ~580 nm (insert in Figure 2b and Figure 3). In europium-containing phases, the ^5^D_0_ → ^7^F_0_ transition is forbidden for electric and magnetic dipole interactions. For this reason, the intensity of this transition is very low or not observed. The number of peaks in the region of the ^5^D_0_ → ^7^F_0_ transition indicates often the number of europium polyhedra in the compound. In the studied scheelites for all the compositions, only one peak is found in the region of the ^5^D_0_ → ^7^F_0_ transition (insert in Figure 2b and Figure 3). This is because the same environment of Eu^3+^ cations in the studied phases, and because europium cations occupy only one polyhedron [33].

In accordance with Table S1, the change of the symmetry from tetragonal for Ag_0.5_*R*_0.2−*y*_Sm*_y_*Eu^3+^_0.3_WO_4_ (*R* = Eu, Gd, Sm) to monoclinic for A*_x_*GS*_y_*E (*x* = 0.286, 0.20; *y*= 0.01, 0.02, 0.03) practically does not change the value of the R/O ratio and the position of the peaks in the region of ^5^D_0_ → ^7^F_0_ transitions (Figure 2b and Figure 3). Figure 4 shows the comparative integral luminescence intensity of the ^5^D_0_ → ^7^F_2_ transition (Eu^3+^) for Ag*_x_*Eu^3+^_(2−*x*)/3_☐_(1−2*x*)/3_WO_4_, Ag*_x_*Sm_(2−*x*)/3−0.3_Eu_0.3_☐_(1−2*x*)/3_WO_4_ and A*_x_*GS*_y_*E (*x* = 0.50, 0.286, 0.20; *y* = 0.01, 0.02, 0.03) (λ_ex_ = 395 nm). According to Figure 4 and Table S1, the luminescence intensity of the ^5^D_0_ → ^7^F_2_ transition is increased from ~2 times (*x* = 0.2, *y* = 0.01 and *x* = 0.286, *y* = 0.02) to ~4 times (*x* = 0.5, *y* = 0.01) during Gd^3+^ and Sm^3+^ co-activation. The integrated emission intensity of Ag_0.20_Gd_0.29_Sm_0.01_Eu_0.30_WO_4_ in the ^5^D_0_ → ^7^F_2_ transition is ~20% higher than that of the Gd_2_O_2_S:Eu^3+^ phosphor, which is used in industry (Figure 5). This confirms that Ag_0.286_Gd_0.252_Sm_0.02_Eu_0.30_WO_4_ and Ag_0.20_Gd_0.29_Eu_0.30_Sm_0.01_WO_4_ with a (3 + 1)D monoclinic incommensurately modulated structure are more attractive for use as red-emitting phosphors for LEDs.

**Figure 9:**
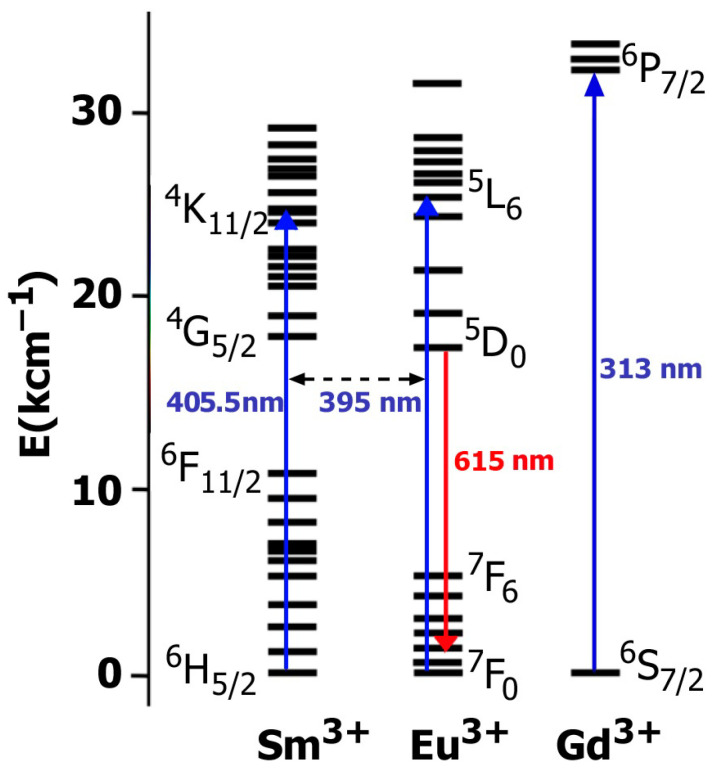
Energy level of Sm^3+^, Eu^3+^ and Gd^3+^ ions. Blue arrows denote the transitions from the ground state to excited levels. Red arrow denotes radiative relaxation from the excited level to the ground state.

However, an increase in Sm^3+^ concentration leads to a noticeable decrease in Eu^3+^ luminescence intensity at the ^5^D_0_ → ^7^F_2_ transition. The intensity of the ^5^D_0_ → ^7^F_2_ transition of the A*_x_*GS*_y_*E (*x* = 0.5, 0.286, 0.2) phases is reduced almost 24 and 73 times with increasing *y* from 0.03 to 0.20 (*x* = 0.5) and 0.30 (*x* = 0.20), respectively. A further increase in the concentration of Sm^3+^ cations leads to lower europium luminescence intensity (Figure 4 and Table S1). The phosphors with a high concentration of Sm^3+^ are not related to the materials with a high luminescence efficiency [34,35] due to the large number of Sm^3+^ energy levels in the visible region of the spectrum (Figure 9). The presence of a large number of energy levels in the Sm^3+^ cation promotes energy transfer between the Sm^3+^ and Eu^3+^ cations. However, the opposite effect of energy transfer to the Sm^3+^ cation is also possible, with further luminescence quenching at defects or due to cross relaxation. On the contrary, when Gd^3+^ cations are introduced into the matrix, the reverse energy transfer to the Gd^3+^ cation is impossible due to the large energy difference (3.96 eV) between the ground and first excited states (Figure 9). Thus, when energy is transferred from Gd^3+^ to Eu^3+^, reverse transfer to the Gd^3+^ cation is impossible. For this reason, the introduction of Gd^3+^ cations into a matrix with europium is more efficient than the introduction of Sm^3+^.

Figure 6 shows the luminescence spectra of Ag_0.20_Gd_0.29_Sm_0.01_Eu_0.30_☐_0.20_WO_4_ as a function of temperature. The luminescence intensity of the ^5^D_0_ → ^7^F_2_ transition in the region of ~615 nm decreases with increasing temperature and completely disappears at T = 473 K. As the temperature increases, the rapid quenching of the luminescence seems to be due to the nonradiative population of the excited states. The temperature dependencies of the luminescence intensity of the ^5^D_0_ → ^7^F_2_ transition at ~615 nm can be compared for the Ag_0.20_Gd_0.29_Sm_0.01_Eu_0.30_☐_0.20_WO_4_ and Ag*_x_R*_((2−*x*)/3)−0.3−*y*_Sm*_y_*Eu^3+^_0.3_☐_(1−2*x*)/3_WO_4_ phases (Figure 7). The observed thermal quenching depends on both the crystal structure of the compounds and the Sm^3+^ concentration. The thermal behavior of PL in Ag_0.20_Gd_((2−*x*)/3)−0.3−*y*_Sm*_y_*Eu^3+^_0.30_☐_0.20_WO_4_ (*y* = 0, 0.01, 0.30) with the incommensurately modulated (3 + 1) D monoclinic structure differs from one of the Ag_0.5_*R*_0.20_Eu_0.30_WO_4_ (*R* = Gd, Sm) phases with the tetragonal *I*4_1_/*a* scheelite structure. The red luminescence of Eu^3+^ (transition ^5^D_0_ → ^7^F_2_) in the monoclinic phases with *y* = 0 and *y* = 0.01 is more thermally stable than that of the tetragonal phases. However, the full substitution of Gd^3+^ by Sm^3+^ (*y* = 0.30) in Ag_0.20_*R*_0.30_Eu_0.30_WO_4_ leads to lower thermal stability of the ^5^D_0_ → ^7^F_2_ luminescence of Eu^3+^ cation.

**Figure 10:**
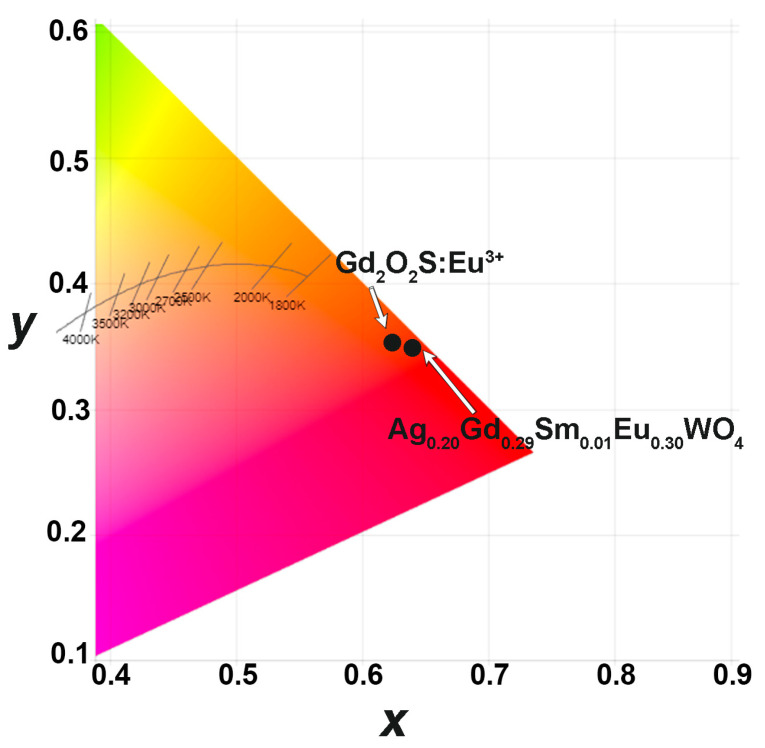
CIE coordinated for Gd_2_O_2_S:Eu^3+^ and Ag_0.20_Gd_0.29_Sm_0.01_Eu_0.30_WO_4_.

The use of synthesized phosphors offers advantages over commercially available Gd_2_O_2_S:Eu^3+^ phosphor. The PL spectrum of Ag_0.20_Gd_0.29_Sm_0.01_Eu_0.30_WO_4_ slightly shifted towards a smaller wavelength (Figure 5). The calculated CIE coordinates (Figure 10) for Gd_2_O_2_S:Eu^3+^ are (0.6312; 0.3562), whereas for Ag_0.20_Gd_0.29_Sm_0.01_Eu_0.30_WO_4_ they are (0.6402; 0.3528). It can be observed from the CIE coordinates that the synthesized phosphor is closer to the red standard of the National Television Standard Committee (NTSC) system (0.67; 0.33). The color purity, calculated by the formula reported in [36], shows the values as 89% for Gd_2_O_2_S:Eu^3+^ and 92% for Ag_0.20_Gd_0.29_Sm_0.01_Eu_0.30_WO_4_. Based on these results, the synthesized phosphors have potential for use in UV-excited LED packages.

## 5. Conclusions

Gd^3+^ and Sm^3+^ co-activation, the effect of cation substitutions and the formation of cation vacancies in Ag*_x_*Gd_((2−*x*)/3)−0.3−*y*_Sm*_y_*Eu^3+^_0.30_☐_(1−2*x*−2*y*)/3_WO_4_ (*x* = 0.5, 0.286, 0.2; *y* = 0.01, 0.02, 0.03) with a scheelite-type structure have been studied here as a factor for managing the luminescence properties. The XRD study of Ag*_x_*Gd_((2−*x*)/3)−0.3−*y*_Eu^3+^_0.30_Sm*_y_*☐_(1−2*x*−2*y*)/3_WO_4_ (*x* = 0.286, 0.2; *y* = 0.01, 0.02, 0.03) shows that its structures have incommensurately modulated character, as well as the structures of the other cation deficient scheelite-related phases. The strongest line at 395 nm in the PLE spectra of the synthesized phosphors is in good agreement with GaN LED chips emitting in the near-ultraviolet range. The electric dipole transition ^5^D_0_ → ^7^F_2_ at ~615 nm has the maximum intensity for all the PL spectra, and its intensity increases from ~2 times (*x* = 0.2, *y* = 0.01 and *x* = 0.286, *y* = 0.02) to *~*4 times (*x* = 0.5, *y* = 0.01) when co-activation with Gd^3+^ and Sm^3+^ is implemented. Ag_0.286_Gd_0_._252_Sm_0.02_Eu_0_._30_WO_4_ and Ag_0_._20_Gd_0_._29_Sm_0.01_Eu_0_._30_WO_4_ with a monoclinic incommensurately modulated (3 + 1)D monoclinic structure are very attractive as phosphor materials for converting near-UV radiation in LEDs. A considerable thermal quenching of the observed luminescence is revealed. The temperature dependence and behavior of the considered phases are influenced by the crystal structure of scheelite-type tungstates and the concentration of Sm^3+^ ions.

## Figures and Tables

**Table 1:** Unit cell parameters and symmetry of Ag*_x_R*_((2−*x*)/3)−0.3_Eu^3+^_0.30_☐_(1−2*x*)/3_WO_4_ (*x* = 0.5, 0.286, 0.2) solid solutions were determined from X-ray diffraction data.

*R*_((2−*x*)/3)−0.3_	*a*, Å	*b*, Å	*c*, Å	γ, Deg.	V, Å^3^	q	*Ref.*
*x* = 0.50 (SG *I*4_1_/*a*)							
Sm_0.20_	5.2780(1)		11.5340(6)		321.31(6)		
Eu_0.20_	5.2787(1)		11.50026(7)		320.482(3)		[20]
Gd_0.20_	5.2740(1)		11.49348(6)		319.689(2)		[20]
*x* = 0.286 (SSG *I*2/*b*(αβ0)00)							
Sm_0.271_	5.2311(2)	5.2950(2)	11.5501(5)	91.460(3)	319.82(3)	0.602a* + 0.817b*	
Eu_0.271_	5.2309(2)	5.2897(2)	11.52995(4)	91.3121(3)	318.948(1)	0.586a* + 0.819b*	[20]
Gd_0.24_Sm_0.03_	5.2188(3)	5.2841(1)	11.5220(5)	91.570(3)	317.62(4)	0.608a* + 0.806b*	
Gd_0.25_Sm_0.02_	5.2153(1)	5.2872(1)	11.5262(2)	91.561(2)	317.71(1)	0.598a* + 0.814b*	
Gd_0.26_Sm_0.01_	5.2169(1)	5.2902(1)	11.5292(2)	91.689(2)	318.05(1)	0.601a* + 0.808b*	
Gd_0.271_	5.2218(2)	5.2880(2)	11.52406(4)	91.4677(4)	318.106(4)	0.585a* + 0.820b*	[20]
*x* = 0.20 (SSG *I*2/*b*(αβ0)00)							
Sm_0.30_	5.2307(1)	5.3003(1)	11.55624(2)	92.0647(2)	320.180(1)	0.591a* + 0.802b*	
Eu_0.30_	5.2193(2)	5.2897(2)	11.52562(4)	92.1118(3)	317.993(1)	0.591a* + 0.800b*	[20]
Gd_0.27_Sm_0.03_	5.2074(2)	5.2858(1)	11.5164(2)	92.345(3)	316.73(2)	0.590a* + 0.801b*	
Gd_0.28_Sm_0.02_	5.2058(1)	5.2856(1)	11.5189(4)	92.345(2)	316.69(2)	0.590a* + 0.805b*	
Gd_0.29_Sm_0.01_	5.2049(1)	5.2839(1)	11.5181(2)	92.374(2)	316.50(1)	0.592a* + 0.803b*	
Gd_0.30_	5.2097(2)	5.2872(3)	11.51483(6)	92.2489(3)	316.929(3)	0.592a* + 0.803b*	[20]

Note: a* and b* are reciprocal parameter vectors.

## Data Availability

Data will be available from the corresponding author on reasonable request.

## Supplementary Materials

The following supporting information can be downloaded at: https://www.mdpi.com/article/10.3390/ma16124350/s1, Table S1: Positions (λ_max_, nm) and integral intensities (*I*_int_) of two maximum bands on PL spectra of Ag*_x_R*_((2−*x*)/3)−0.3−*y*_Sm*_y_*Eu^3+^_0.3_☐_(1−2*x*)/3_WO_4_ (*R* = Eu, Gd, Sm; *x* = 0.50, 0.286, 0.20; *y* = 0, 0.01, 0.02, 0.03) scheelite-type phases. All samples are measured under the same conditions.
