# Supplementary material for: Novel Red Phosphor of Gd3+, Sm3+ co-Activated AgxGd((2−x)/3)−0.3−ySmyEu3+0.30☐(1−2x−2y)/3WO4 Scheelites for LED Lighting
Source: Materials (Basel). 2023 Jun 13;16(12):4350. doi: 10.3390/ma16124350 (PMC10303245; doi:10.3390/ma16124350)
Supplement: Supplementary file 1 [file materials-16-04350-s001.zip › materials-2430200-supplementary.pdf]

Supporting Information

Novel red phosphor of Gd^{3+}, Sm^{3+} co-activated $Ag_xGd_{((2-x)/3)-0.3-y}Sm_yEu^{3+}_{0.30}\square_{(1-2x-2y)/3}WO_4$ Scheelites for LED Lighting

Vladimir Morozov [1], Bogdan I. Lazoryak [1], Aleksandra A. Savina [2,3], Elena G. Khaikina [3], Ivan I. Leonidov [4], Alexey V. Ishchenko [5], Dina Deyneko [1,6],*

[1] Chemistry Department, Moscow State University, 119991 Moscow, Russia; morozov111vla@mail.ru (V.A.M.); bilazoryak@gmail.com (B.I.L.)
[2] Skolkovo Institute of Science and Technology, 121205, Moscow, Russia; a.savina@skoltech.ru
[3] Baikal Institute of Nature Management, Siberian Branch, Russian Academy of Science, Ulan-Ude 670047, Russia
[4] Institute of Solid State Chemistry, Ural Branch, Russian Academy of Sciences, Ekaterinburg 620990, Russia
[5] NANOTECH Center, Ural Federal University, Ekaterinburg 620002, Russia
[6] Laboratory of Arctic Mineralogy and Material Sciences, Kola Science Centre, Russian Academy of Sciences, 184209 Apatity, Russia
* Correspondence: deynekomsu@gmail.com

Table S1. Positions (λ_{max}, nm) and integral intensities (I_{int}) of two maximum bands on PL spectra of $Ag_xR_{((2-x)/3)-0.3-y}Sm_yEu^{3+}_{0.3}\square_{(1-2x)/3}WO_4$ (R = Eu, Gd, Sm; x = 0.50, 0.286, 0.20; y= 0, 0.01, 0.02, 0.03) scheelite-type phases. All samples are measured under the same conditions.

Phase	$^5D_0 \rightarrow {}^7F_0$	$^5D_0 \rightarrow {}^7F_1$	$^5D_0 \rightarrow {}^7F_2$			^{a}R/O	$^bI_{int}/I_0$
	λ_{max}	I_{int}	λ_{max1}	λ_{max2}	I_{int}		
		x = 0.50					
$Ag_{0.50}Eu_{0.20}Eu_{0.30}WO_4$	580	34	612.4	615.5	301	8.8	1.0
$Ag_{0.50}Gd_{0.20}Eu_{0.30}WO_4$	580	52	612.4	615.5	457	8.8	1.5
$Ag_{0.50}Gd_{0.19}Sm_{0.01}Eu_{0.30}WO_4$	580	218	612.4	615.5	1860	8.5	**6.2**
$Ag_{0.50}Gd_{0.18}Sm_{0.02}Eu_{0.30}WO_4$	580	175	612.4	615.5	1492	8.5	5.0
$Ag_{0.50}Gd_{0.17}Sm_{0.03}Eu_{0.30}WO_4$	580	173	612.4	615.5	1438	8.5	4.8
$Ag_{0.50}Sm_{0.20}Eu_{0.30}WO_4$	580	16	612.4	615.5	135	8.8	0.4
		x = 0.286					
[1]$Ag_{0.286}Eu_{0.272}Eu_{0.30}WO_4$	580.2	17	612.7	615.7	149	8.8	0.5
$Ag_{0.286}Gd_{0.272}Eu_{0.30}WO_4$	580.2	172	612.8	615.6	1512	8.8	5.0
$Ag_{0.286}Gd_{0.262}Sm_{0.01}Eu_{0.30}WO_4$	580.2	260	612.9	615.6	2181	8.4	7.2
$Ag_{0.286}Gd_{0.252}Sm_{0.02}Eu_{0.30}WO_4$	580.2	308	612.9	615.6	2609	8.5	**8.7**
$Ag_{0.286}Gd_{0.242}Sm_{0.03}Eu_{0.30}WO_4$	580.3	199	612.8	615.6	1630	8.2	5.4
$Ag_{0.286}Sm_{0.272}Eu_{0.30}WO_4$	580.2	3	612.7	615.5	31	9.1	0.1
		x = 0.20					
[1]$Ag_{0.20}Eu_{0.30}Eu_{0.30}WO_4$	580.1	16	612.8	615.7	140	8.5	0.5
$Ag_{0.20}Gd_{0.30}Eu_{0.30}WO_4$	580.3	163	613.0	615.5	1425	8.7	4.7
$Ag_{0.20}Gd_{0.29}Sm_{0.01}Eu_{0.30}WO_4$	580.4	333	613.0	615.5	2832	8.5	**9.4**
$Ag_{0.20}Gd_{0.28}Sm_{0.02}Eu_{0.30}WO_4$	580.4	300	613.0	615.5	2571	8.6	8.5
$Ag_{0.20}Gd_{0.27}Sm_{0.03}Eu_{0.30}WO_4$	580.3	262	613.0	615.5	2211	8.4	7.3
$Ag_{0.20}Sm_{0.30}Eu_{0.30}WO_4$	-	3	613	615.5	21	8.5	0.1

[1] The compositions determined by the crystal structure refinements are $Ag_{0.238}Eu_{0.587}\square_{0.175}WO_4$ and $Ag_{0.157}Eu_{0.614}\square_{0.229}WO_4$, respectively.

^{a} R/O is the $I_{int}(^5D_0 \rightarrow {}^7F_2)/I_{int}(^5D_0 \rightarrow {}^7F_1)$ ratio, often referred to as the asymmetry ratio.

^{b} I_{int} and I_0 are the integral intensity for the $^5D_0 \rightarrow {}^7F_2$ transition on PL spectra of the studied phase and $Ag_{0.50}Eu_{0.50}WO_4$, respectively.
